# Visual Perspectives in Episodic Memory and the Sense of Self

**DOI:** 10.3389/fpsyg.2018.02196

**Published:** 2018-11-13

**Authors:** Ying-Tung Lin

**Affiliations:** Institute of Philosophy of Mind and Cognition, National Yang-Ming University, Taipei, Taiwan

**Keywords:** episodic memory, episodic simulation, observer perspective, visual perspective, self-consciousness, phenomenal presence of self, sense of identity

## Abstract

The connection between memory and self-consciousness has been a central topic in philosophy of memory. When remembering an event we experienced in the past, not only do we experience being the subject of the conscious episode, but we also experience being the protagonist in the memory scene. This is the “phenomenal presence of self.” To explore this special sense of self in memory, this paper focuses on the issue of how one identifies oneself in episodic simulation at the retrieval of memory and draws attention to the field and observer perspectives in episodic memory. Metzinger (2013a,b, 2017) recently introduced the concept of the phenomenal unit of identification (UI) to characterize the phenomenal property that gives rise to the conscious experience of “I am this.” This paper shows how observer-perspective remembering provides an interesting opportunity for studying the sense of self. It is argued that observer-perspective remembering is a stable state of consciousness that is distinct from autoscopic phenomena with respect to the dimensions of minimal phenomenal self (MPS). Together, the notion of UI and the particular style of remembering offer a way of understanding the phenomenal presence of self, and three possible ways in which phenomenal properties constitute UI in memory are raised. The study of perspectives in episodic simulation may prompt new empirical and conceptual issues concerning both the sense of identity and the relationship between MPS and extended self.

## Introduction

When remembering an event we experienced in the past, a special sense of self is involved: not only do we experience being the subject of the conscious episode, but we also experience being the protagonist in the memory scene. That is, the one witnessing in the present and the one in the past who is witnessed are experienced as one and the same person. This is the “phenomenal presence of sel” (Rowlands, 2017). How does one identify oneself in episodic simulation during the retrieval of autobiographical memory?

The connection between memory and the sense of self has been a central topic in philosophy of memory; the role of memory in constituting personal identity, psychological self, and narrative has long been a major field of enquiry (Schechtman, 1996; Locke, 2008; Klein and Nichols, 2012; Klein, 2014; Hutto, 2017). Recently, more attention has been paid to self-awareness (Thompson, 2010), self-concept (Klein, 2012; Rowlands, 2017), and immunity (Fernández, 2014; Bermudez, 2017) in memory. However, I believe the issue of what constitutes the phenomenal presence of self remains underexplored. This paper aims to address it.

One important concept is that of “the phenomenal unit of identification (UI),” which has been recently introduced by Metzinger (2013a,b, 2017) to characterize the phenomenal property that gives rise to the conscious experience of “I am this.” Candidates of the phenomenal property in question are the dimensions of the minimal phenomenal self (MPS), and altered states of consciousness – such as out-of-body experiences, full-body illusions, dreams, and mind-wandering – have been critical to our understanding of them (Metzinger, 2004, 2013b, 2017; Blanke and Metzinger, 2009; Windt, 2015). This paper aims to bring attention to field and observer perspectives in episodic memory and to suggest that studying these phenomena may provide an interesting approach to researching the phenomenal presence of self and UI in memory.

The paper begins by introducing the phenomenon of perspective shifting, and the target question of what constitutes the phenomenal presence of self is then elaborated. Next, I argue that observer-perspective remembering is a stable state of consciousness that is distinct from autoscopic phenomena with respect to the dimensions of MPS and as such can provide an interesting approach to researching identification in memory and its degree. The paper also notes that studying perspectives in episodic memory can draw out empirical and conceptual issues concerning the relationship between the MPS and extended self.

It is worth noting that “memory” is an equivocal term^1^ and is used to refer to retrieved information in this paper. In addition, the discussion here is restricted to “episodic memory,” a kind of declarative memory whose content is a reconstruction of an event in the past with temporal, spatial, and self-referential context (Tulving, 1983; Klein, 2015). Phenomenally, we experience as if we mentally travel back in time and “re-live” an event we once experienced.^2^ Moreover, memory is regarded as a kind of conscious or episodic simulation. According to “constructive memory framework” Schacter et al. (1998), representations of experiences are conceptualized as “patterns of features,” which represent different facets of the experience. Retrieval is realized by the process of pattern completion, which allows a subset of features to comprise a past experience (Schacter, 1999, 2001; Schacter and Addis, 2007; Schacter et al., 2011).^3^ It is suggested that the mechanism of memory construction is shared by other mental phenomena including future and counterfactual thinking (Buckner and Carroll, 2007; Schacter et al., 2008; De Brigard, 2013).

## Field and Observer Perspectives in Episodic Simulation

I see myself dancing at a party at the university. I remember my clothes and my legs (the way they moved). Suddenly, I am “inside my own body” looking out. A guy I know a little walks by me and says as he passes: “You look good today” (Berntsen and Rubin, 2006, p. 1193).

The experience of visual images is commonly involved when remembering an event, and two perspectives can be adopted in recollection: field and observer. When one recalls something from a field perspective, the event is viewed from the visual first-person perspective of the represented self. The visuospatial image originates from the same viewpoint experienced at encoding. In contrast, someone remembering can take an observer perspective in which an event is visualized from an external vantage point. Observer perspectives are located outside one’s represented body and can vary across spatial locations depending on several factors. In the memory report above, the subject first experienced the recollection from an observer perspective and then shifted to a field perspective.

Despite the difficulty of pinning down the actual proportion of recollections from an observer perspective to those from a field perspective, it has been suggested that the observer perspective may be more common than initially thought (Rice and Rubin, 2011). A cross-cultural study of the prevalence of the two visual vantage points in imaginary events during mind-wandering showed that almost half (46%) of participants reported most commonly adopting a third-person visual perspective (Christian et al., 2013). Additionally, McDermott et al. (2016) revealed that future events are more likely to be imagined from an observer perspective. Although the tendency to adopt an observer perspective may be different in memory, these studies indicate that observer perspectives can be found in a significant minority of mental events.

Recently, the topic of visual perspective in memory has gained attention in cognitive psychology. Studies of visuospatial perspectives have focused on its effects on emotional intensity (Berntsen and Rubin, 2006), recollection content (Mcisaac and Eich, 2002), personal continuity (Libby and Eibach, 2002; Libby et al., 2005), self-projection (D’Argembeau and Van Der Linden, 2004), the truthfulness (or accuracy) of memory (Heaps and Nash, 2001), and the links to clinical disorders such as post-traumatic stress disorder (Porter and Birt, 2001; Berntsen et al., 2003; Mcisaac and Eich, 2004) and body dysmorphic disorder (Osman et al., 2004). Rice and Rubin (2009) studied the relationship between the field and observer perspectives in a given episode of memory. Can we experience a single recalled experience from more than one vantage point? A mutually exclusive framework, which is presumed in many studies of perspective in memory (e.g., Nigro and Neisser, 1983), suggests that we only experience either a field or an observer perspective, but not both, in a single retrieval attempt. However, complementary and independent frameworks propose that both perspectives can be experienced. More adoption of one perspective necessitates lesser adoption of the other according to the complementary framework, whereas the two perspectives are independent from each other in the independent framework. How their relationship is conceptualized determines how perspective in memory should be assessed. Using different kinds of measurement techniques and examining how two perspectives correlate with ratings of vividness, Rice and Rubin (2009) provide evidence in support of the independent framework.

## The Phenomenal Presence of Self in Episodic Memory

Every episodic memory – with either a field or observer perspective – entails a sense of self (Tulving, 1985; Klein, 2012, 2015).^4^ Each puts one – the subject who is remembering – in contact with an event that involves oneself – who is remembered – and leads to the question: how does one identify oneself in episodic simulation during the retrieval of autobiographical memory? To illustrate the issue, try to recall a public event in the past, such as a recent family reunion. It is very likely that you recall the event from a field perspective (if not, please shift to a field perspective for now; I will refer to this as “field-perspective remembering”). How do you identify yourself among the other people represented in your memory? One may find this question absurd when considering a case in which one remembers an event from a field perspective. You “re-experience” from the vantage point of your simulated eyes, similar to the original experience during encoding; you are probably also embodied in a simulated body. The egocentric center coincides with your body’s location. Let us then try to remember an event from an observer perspective. (Feel free to recall any event as long as the experience originates from an observer’s vantage point. It will be referred to here as “observer-perspective remembering.”) Again, several people are represented in your episodic recollection as if they appear in your visual field, but this time, one of them is you. What enables you to successfully identify yourself?

Compare the experience of observer-perspective remembering with spotting yourself in a group photo. They differ in several ways: first, you probably recognize yourself in the photo through your face, hairstyle, an old item of clothing, or other external properties or combination of properties. However, in observer-perspective remembering, even sometimes without representations of recognizable features that could allow identification,^5^ that person is taken to be yourself in a direct and immediate manner. Additionally, subjectively, you do not experience many – if any – differences in the way of identification when remembering from an observer perspective when compared with field-perspective remembering. Second, when recognizing yourself in a photo, you may doubt if the person in that image was really you; however, there is no such doubt in autobiographical remembering. Third, such identification is robust in all experiences of episodic autobiographical memory. It is considered necessary for episodic memory by Tulving (1983, 1985), who regarded remembering as an expression of autonoetic consciousness.

The identification with these features is also present in some forms of future thinking, imagining, mind wandering, and dreams (Rosen and Sutton, 2013). Addressing the issue of identification, Metzinger (2013a,b, 2017) introduced the concept of the phenomenal UI to characterize the phenomenal property that gives rise to the conscious experience of “I am this” – that is, the phenomenal presence of self. I will examine identification and UI in memory with an observer perspective after the next section, in which I argue that observer-perspective remembering is itself an interesting phenomenon with respect to the dimensions of MPS.

## Dimensions of MPS in Memory

What is the minimal form of self-consciousness and what are its enabling conditions? Many studies of MPS and bodily self-consciousness have focused on autoscopic phenomena, particularly out-of-body experiences. Three dimensions of MPS are proposed to characterize different forms of autoscopic phenomena: self-location, self-identification, and weak first-person perspective (weak 1PP) (Blanke and Metzinger, 2009). Self-location refers to being located at a spatiotemporal point or space that in a standard situation would be localized within one’s represented body; self-identification is the global identification of the body as a whole; and weak 1PP is the geometrical origin of an egocentric visuospatial model of reality.

These concepts enable clear characterizations of autoscopic phenomena such as autoscopic hallucination, heautoscopy, and out-of-body experiences (Blanke and Mohr, 2005; Blanke and Metzinger, 2009). People with autoscopic hallucinations experience seeing an illusory double of their own body in an extrapersonal space, while their visuospatial perspective (weak 1PP) and the location of their experience of embodiment remain unchanged (self-location and self-identification). During out-of-body experiences, one’s self-location and weak 1PP are located outside one’s physical body, whereas one’s identification with the physical body is lost and instead one identifies with an illusory body. Heautoscopy is considered an intermediate form between autoscopic hallucinations and full out-of-body experiences. Individuals with heautoscopy, experiencing an illusory body and an extrapersonal space, are unable to determine their self-location and self-identification, and the origin of their weak 1PP is reported to either alternate between the real and illusory bodies or exist at both locations simultaneously. Recently, bodiless experiences – such as asomatic out-of-body experiences and bodiless dreams – have been invoked to argue against the necessity of self-identification for phenomenal minimal selfhood and to suggest that only the experience of spatiotemporal self-location is required (Windt, 2010; Metzinger, 2013b).

One essential difference between remembering and autoscopic phenomena is worth noting before examining three dimensions of MPS in episodic memory. Regarding remembering, having episodic memory means having a perception-like experience: a virtual world is mentally simulated in which one’s virtual body is situated. In contrast, an illusory body is experienced in extrapersonal space in the real world in autoscopic phenomena. Additionally, unlike autoscopic phenomena, remembering involves the experience of temporal self-location in the past, which contributes to autonoetic consciousness. Autonoetic consciousness allows one to mentally project oneself backward to experienced events and provides the familiar phenomenal flavor of recollective experience characterized by “pastness” (Tulving, 1983, 1985).

How do self-location, self-identification, and weak 1PP appear in remembering? During recollection from a field perspective, in one’s episodic simulated world, three dimensions of MPS coincide and are localized within the boundaries of one’s virtual body. In contrast, while recalling an event from an observer perspective, the origin of our visuospatial perspective is by definition located outside the virtual body; its location depends on the content of recollection and several other factors (Rice, 2010). But what about self-identification and self-location?

It may be conjectured that decoupled visuospatial perspective results in reduced self-identification; however, some sports psychology studies challenge this idea. Perspectives are important in the inquiry of how imagery perspectives affect the performance of a given skill. It was assumed that expertise in sports is associated with increased use of internal imagery (i.e., imagery from a field perspective; e.g., Mahoney and Avener, 1977) and that kinesthetic imagery – i.e., the simulation of the somatosensory consequences of imagined movement such as proprioception (Stinear, 2010) – can only, or will be more easily, be performed with an internal image (e.g., Hale, 1982). However, Dana and Gozalzadeh (2017) found external imagery more effective for open forms of skill performance that include “changing environmental conditions, intertrial variability, body transport, and object manipulation” (p. 4) in tennis (e.g., forehand accuracy), but internal imagery for closed forms (e.g., serve accuracy). Furthermore, Callow and Hardy (2004) examined the strength of the relationship between visual imagery and kinesthetic imagery and found no significant correlation when subjects were instructed to imagine watching someone else from an external perspective. Nevertheless, there was a significant correlation between external and kinesthetic imagery when the subjects were instructed to imagine “watching yourself” from an external perspective, but not between internal and kinesthetic imagery. The authors speculate that the correlation was due to the form of execution involved in this study, which requires greater “spatial positioning of movement” and “visual referencing of its location.” These studies suggest that a dissociated weak 1PP can give rise to stronger identification with the virtual body in certain situations.

As for self-location, it remains unclear where it is; this is surely a question open to experimental investigation. According to the aforementioned studies, open forms of skill performance benefit more from observer-perspective imagery and this type of skill involves monitoring constantly changing environmental factors and their spatial relationships to external objects. It is suggested that the experience from an observer perspective can lead to a more accurate visuospatial self-location – where accuracy refers to the degree to which the spatial relation between self-location and virtual body in episodic simulation corresponds to the spatial relation between self-location and physical body or objects in reality, and that such effect may also be found in memory. Self-location is predicted to remain within the boundary of one’s body in observer-perspective remembering.

If the analysis is correct, remembering with an observer perspective is a phenomenon in which one’s weak 1PP is decoupled from self-identification and self-location, which is hardly found in autoscopic phenomena. Given that such decoupling is also present in dreams (Rosen and Sutton, 2013), observer-perspective remembering can serve as a stable state of consciousness that is distinct from autoscopic phenomena and can be easily manipulated and assessed.

## Identification in Memory With an Observer Perspective

Returning to the question of how we identify ourselves in memory (as well as in future thinking and dreams) with an observer perspective, UI – defined as the phenomenal property that gives rise to the conscious experience of “I am this” – can be linked to a range of different phenomenal properties and used to characterize various states of consciousness such as mind-wandering (Metzinger, 2013a, 2017) and dreams (Metzinger, 2013b). According to UI-theory (Metzinger, 2013b), UI can change dynamically as we “constantly search for a source of maximal invariance” (p. 5) and identify with it. In a standard situation in which we experience ourselves as embodied agents, UI candidates are contents of our body as a whole and the agentive experience of being in control of one’s bodily actions (Metzinger, 2013a, p. 10), whereas when we are experienced as an epistemic agent, UI is the “epistemic agent model” (EAM) defined as a conscious self-representation of being equipped with epistemic self-control (e.g., maintaining knowledge relation to certain parts of the real/virtual world, body and oneself) (Metzinger, 2013b, 2015). In many cases, including observer-perspective remembering, UI can be a combination of these.

The concept of UI allows us to characterize episodic memory with field and observer perspectives. Like mind-wandering (Metzinger, 2013a), every beginning and ending of an episodic autobiographical simulation – from a real world- and self-model to a virtual (past) world- and self-model or back – is a shift of the UI. However, unlike mind-wandering, which comprises causally determined unconscious processes, episodic simulation can also be deliberately initiated, and UI shifts are therefore not necessarily accompanied by a brief loss of self-awareness, i.e., a “self-representational blink” (SRB) (Metzinger, 2013a). Furthermore, there may be more UI shifts in a memory episode.

The issue here is whether the UI shifts when one changes the vantage point from which one recollects – from field to observer perspective, or vice versa. In this respect, we might ask: what constitutes UI when remembering from an observer perspective? To account for UI in this special case in which the origin of weak 1PP is decoupled from self-location and self-identification, three options are available.

The first possibility is to identify with the “observer” – for UI to coincide with either a visuospatial perspective or an EAM. This is intuitive when considering self-references in the memory report such as “I see myself dancing at a party at the university” (Berntsen and Rubin, 2006, p. 1193). The first-person pronoun refers to the “observer” as an epistemic agent. In this case, UI switches from an embodied agent to a disembodied epistemic agent when changing one’s vantage point from field to observer perspective. However, one can also identify with the “protagonist”; here, the UI can be the content of the virtual body image or sense of bodily agency. The finding that observer-perspective imagery is only significantly associated with kinesthetic imagery when subjects imagine watching themselves (Callow and Hardy, 2004) suggests that we may experience ourselves identifying with the embodied agent, and no UI switching accompanies visual perspective shifting.

Consider the third possibility: UI is constituted by both the phenomenal embodied agency and epistemic agency, as well as the relation between these two, such as the spatial relation (e.g., distance, in front/behind, and height). Rice and Rubin (2011) found that the location of the vantage point from which one remembers from an observer perspective is reliably associated with the events being recalled. For instance, remembering a face-to-face conversation and a group performance, respectively, produced images near the protagonist and images from a distance, whereas remembering giving an individual presentation and remembering being in an accident, respectively, produced images from a perspective in front of a protagonist and images from behind.

To be more specific, in observer-perspective remembering, if the relationship between the observing agent and embodied agent – the protagonist – contributes to the UI, it is hypothesized that there is a connection between the relationship and the degree of UI. No empirical study of which I am aware has assessed such a connection. However, if we consider remembering from a field perspective as one extreme of such a relationship, some currently available data indirectly supports such a hypothesis. The connection between perspectives and the personal assessment of self-change has been studied by Libby and Eibach (2002) and Libby et al. (2005). They found that compatibility between one’s current self-concepts (e.g., religious beliefs, political attitudes, and the nature of their relationships) and the actions visualized in memory affect the vantage points from which the subjects view a scene: conflicting actions tend to be viewed from an observer perspective, while compatible actions are viewed from a field perspective (Libby and Eibach, 2002). UI in remembering offers an interesting perspective on understanding the phenomenal presence of self in memory and one’s sense of identity, which I will illustrate next.

## The Degree of Phenomenal Presence of Self and the Sense of Identity

One implication of the third conceptual possibility – whereby UI is partially constituted by the relationship between the epistemic agent and the embodied agent in memory – is that our phenomenal sense of self can emerge in degrees. Factors involved in determining the degree of identification may affect the relationship in question. There are a number of trait and state differences figured into the degree of identification. Regarding trait differences, some individuals are more capable of forming UI than others (D’Argembeau and Van Der Linden, 2006). Meanwhile, state differences illustrate the different likelihoods of a possible model being integrated into the current one. Some possible models are more likely to be integrated than others by the current self-model. The difference is largely dependent on the system’s current state and the compatibility between the autobiographical and emotional content of the current state and a given possible state. As a system constantly aims to maximize its coherence, a form of integration that allows “distance” between one’s current and past (or future) self-models is available to enable successful integration; in this case, remembering (or future thinking) from an observer perspective is accompanied by a reduced sense of identity. On the other hand, in some specific cases in which information obtained from an observer perspective (e.g., one’s location in a group) is required for or can boost successful integration, observer-perspective remembering may bring one enhanced sense of identity.

This view resonates with the notion of “self-distancing” in cognitive therapy (Kross and Ayduk, 2016). The process of self-distancing refers to a mechanism that allows subjects to analyze their past experience from a self-distanced perspective instead of an immersive perspective. The underlying idea is that cueing subjects to analyze negative experiences from an immersive perspective will lead them to focus on the emotionally arousing feature of the experience, whereas taking a self-distanced perspective will shift the focus more to reconstructing the episode in ways that offer a sense of insight and closure. Shifting visual perspectives is a common technique: subjects are asked to “take a few steps back” and to watch the experience happening to them “from the vantage point of a fly on the wall” (observer-perspective episodic simulation; Kross and Ayduk, 2016, p. 87). There are different types of distancing domains (e.g., non-first-person self-talk in the linguistic domain) and all are connected inasmuch as enhancing distance in one domain results in the enhancement of distance in other domains.

Furthermore, the degree of UI in memory – as the experience of the degree to which one identifies with the simulated past self – can be taken to understand the sense of identity or continuity. It should be noted that there is a distinction between sense of identity (or sense of continuity) and the metaphysical relationship of identity; only the former is of concern here. Unlike the metaphysical relationship, a sense of identity is a phenomenological concept: as we experience ourselves not only as existing at the present moment, but also in the past and potentially in the future, the concept refers to our feeling that we are a temporally extended being (cf. the autobiographical self in Damasio, 1999). The point here is to point out that studying perspectives and dimensions of MPS in episodic simulation (remembering, future, and counterfactual thinking) can open up new avenues for understanding the connection between the MPS and the extended self (Gallagher, 2000).

## Conclusion

Together, the notion of UI and the special case of observer-perspective remembering offer a way of understanding the phenomenal presence of self in episodic memory and how it is linked to our sense of identity. I have shown how the particular style of remembering is relevant to research on phenomenal selfhood and identification in memory. The analysis can be further extended to study future thinking and vicarious dreams (Rosen and Sutton, 2013) and can inform issues ranging from theoretical inquiry in philosophy of memory to clinical applications in cognitive therapy.

## Author Contributions

The author confirms being the sole contributor of this work and has approved it for publication.

## Conflict of Interest Statement

The author declares that the research was conducted in the absence of any commercial or financial relationships that could be construed as a potential conflict of interest.

## Footnotes

1 The term “memory” may refer to neurocognitive capacity, hypothetical store, information persistence, information retrieval, or the phenomenal awareness of remembering (Tulving, 2000).

2 Note that “re-living” is used in a loose sense here, as memory distortion and perspective switching is allowed. In agreement with Sutton (2010), constructive memory and memory with an observer perspective are considered genuine.

3 The construction of memory is constrained by different factors, including the organism’s present goal (Conway, 2005), the current environment (Anderson and Schooler, 1991), and prior knowledge (Hemmer and Steyvers, 2009).

4 However, it does not imply that semantic memory cannot be self-referential or about oneself. Contextually dependent personal information can reside in semantic memory: “personal semantic memory” (Kopelman et al., 1989).

5 The absence of recognizable features may be due to decreased vividness or accuracy of memory retrieval (Marcotti and St. Jacques, 2018) or the vantage point adopted, which prevents some or any external properties represented in the visual scene.
